# pH-driven spontaneous recovery of tyrosine *via* co-precipitation in the indirect aqueous carbonation of gypsum

**DOI:** 10.1039/d5ra09358a

**Published:** 2026-02-17

**Authors:** Yujie Qu, Yuan Gong, Baizhi Wu, Chunlei Li, Lanying Wang, Yi Wang

**Affiliations:** a School of Petrochemical Technology, Lanzhou University of Technology Lanzhou 730050 PR China yuangong@lut.edu.cn; b Gansu Engineering Research Center for Resource Utilization of New Energy Industry Waste and Waste-Utilized Materials Lanzhou 730050 PR China; c Jiuquan Iron and Steel (Group) Co., Ltd. Jiayuguan 735100 PR China

## Abstract

Efficient recycling of leaching additives is crucial for the economic viability of indirect aqueous carbonation of gypsum. This study presents a pH-driven spontaneous co-precipitation strategy employing tyrosine (Tyr) as a recyclable additive. The method exploits the inherent pH decrease during carbonation to spontaneously precipitate and separate Tyr from the SO_4_^2−^-rich carbonation mother liquor. In a strongly alkaline medium, fully deprotonated Tyr chelates with Ca^2+^, significantly enhancing the solubility of CaSO_4_·2H_2_O. Response surface methodology based on a Box–Behnken design was applied to optimize the leaching conditions, which were determined as follows: Tyr/CaSO_4_·2H_2_O = 5.51 mol mol^−1^, KOH/Tyr = 2.00 mol mol^−1^, and liquid-to-solid ratio = 51.38 mL g^−1^. Under these conditions, the leached Ca^2+^ concentration reached 16.09 ± 0.30 g L^−1^—six times higher than the solubility of CaSO_4_·2H_2_O in pure water at 30 °C (∼2.6 g L^−1^). During the early stage of carbonation, the Ca^2+^–Tyr complexes dissociate as the pH decreases, releasing free Ca^2+^ for direct precipitation of homogeneous calcite, while neutral Tyr^0^ co-precipitates efficiently. Under the optimized carbonation conditions (30 °C, CO_2_ flow rate 150 mL min^−1^, 50 min), the carbonation efficiency and Tyr recovery efficiency exceeded 97% and 95%, respectively. The process also demonstrated excellent stability over five leaching–carbonation cycles, with average values of 12.63 ± 0.56 g L^−1^ for Ca^2+^ leaching concentration, 93.41 ± 1.85% for carbonation efficiency, and 92.73 ± 0.52% for Tyr recovery efficiency.

## Abstract 2

A pH-driven spontaneous co-precipitation strategy enables additive recovery in indirect aqueous carbonation of gypsum. The tyrosine-mediated process achieves >92% recovery and stable cycling over multiple runs.

## Graphical abstract

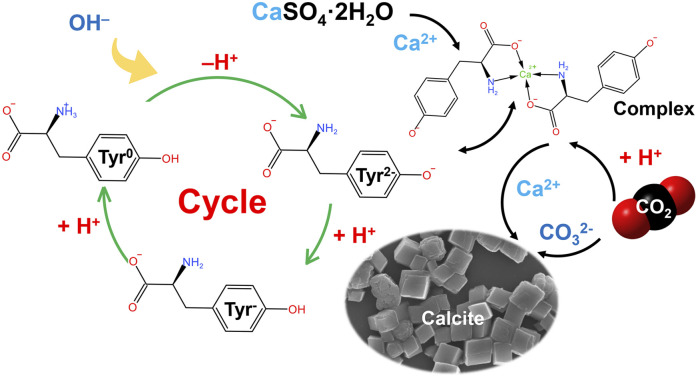

## 1. Introduction

Aqueous carbonation is widely regarded as a promising process for CO_2_ sequestration owing to its favorable thermodynamics, mild operating conditions, and high reactivity.^1,2^ Industrial waste gypsum, primarily composed of CaSO_4_·2H_2_O with a theoretical CaO content of ∼32.5%, serves as an ideal calcium source for this process.^3^ Its use as a substitute for natural calcium-rich minerals therefore offers a synergistic strategy to address both solid waste management and CO_2_ utilization/storage challenges.^4–6^

Aqueous carbonation can be classified into direct and indirect pathways.^7^ The indirect two-step route involves first leaching Ca^2+^ from the solid feedstock, then raising the pH to an alkaline range and introducing CO_2_ into the leachate to precipitate CaCO_3_. Compared with direct carbonation, this approach effectively removes insoluble impurities, operates under milder conditions, and allows precise control over the purity, polymorph, and particle size distribution of the resulting CaCO_3_. These advantages make it a promising route for the high-value utilization of solid waste gypsum.^8,9^ The efficiency and selectivity of calcium leaching are critical, as they directly govern the subsequent carbonation efficiency and the purity of the final carbonate product. Various leaching agents have been explored to enhance leaching performance. For example, Rahmani *et al.*^10,11^ used H_2_SO_4_ to leach Ca^2+^ from red gypsum, while Ding *et al.* employed inorganic salts such as CH_3_COONH_4_, NaCl, and NH_4_Cl to improve the dissolution of desulfurization gypsum or phosphogypsum *via* the salt effect.^12–15^ However, despite their efficacy, the stable recycling of these leaching agents remains a major practical challenge.^4^ In particular, directly recycling the carbonation mother liquor containing additives in a leaching–carbonation cycle can lead to a gradual decline in Ca^2+^ leaching yield due to the common-ion effect from accumulating SO_4_^2−^.^13^ Hence, developing efficient methods to separate and recycle leaching additives from SO_4_^2−^-enriched carbonation mother liquor is crucial for advancing the indirect aqueous carbonation of gypsum.

In this context, organic additives such as amino acids have emerged as promising candidates due to their unique recyclability. Their distinctive molecular structure, featuring both amino and carboxyl groups, enables effective chelation of Ca^2+^, thereby facilitating the dissolution of calcium-based feedstocks.^16^ Recently, amino acids have attracted considerable interest as promoters for the indirect aqueous carbonation of fly ash.^17–19^ Moreover, during carbonation, the amino groups contribute to CO_2_ absorption by forming carbamate and protonated amino acid intermediates. These species hydrolyze upon CaCO_3_ precipitation, regenerating the amino acids with an efficiency surpassing conventional thermal regeneration.^20^ This regenerative mechanism is synergistically driven by the exothermicity of carbonation and the precipitation of CaCO_3_ itself, offering a promising route to reduce the energy consumption and cost associated with amino acid recovery.^21,22^

As is well known, amino acids exhibit minimal solubility at their isoelectric points (pI). This property suggests that selecting amino acids with a strong solubility contrast near their pI could enable their effective separation from SO_4_^2−^-rich carbonation mother liquor, positioning them as candidate additives for indirect gypsum carbonation. In our previous work, using aspartic acid (Asp) as a leaching additive increased the solubility of CaSO_4_·2H_2_O to 19.50 g L^−1^ at 30 °C, roughly ten times higher than in pure water. Importantly, both the additive and SO_4_^2−^ were effectively separated from the mother liquor at the pI of Asp (2.77), enabling stable recycling with a recovery efficiency exceeding 80% over 10 cycles.^16^ However, the low pI of Asp necessitates excess H_2_SO_4_ for pH adjustment, which complicates the regeneration process.

This work presents a strategy for the indirect aqueous carbonation of gypsum using tyrosine (Tyr) as a leaching additive. The approach leverages the finding that Tyr co-precipitates with CaCO_3_ during carbonation, allowing spontaneous separation of the additive from SO_4_^2−^ in the mother liquor without additional acidification. The co-precipitated Tyr is then separated from CaCO_3_ by alkaline washing, and the resulting filtrate, containing redissolved Tyr, is recycled for subsequent Ca^2+^ leaching. To maximize the Ca^2+^ leaching yield, dissolution conditions for CaSO_4_·2H_2_O in the alkaline Tyr–KOH–H_2_O system were first optimized using response surface methodology (RSM). The effects of CO_2_ flow rate, reaction time, and temperature on carbonation efficiency and Tyr recovery were systematically investigated, leading to a proposed mechanism for the Tyr-mediated carbonation process. Finally, cyclic stability was evaluated over five consecutive leaching–carbonation cycles by monitoring Ca^2+^ leaching yield, carbonation efficiency, and Tyr recovery efficiency.

## 2. Materials and methods

### 2.1. Materials

Analytical-grade CaSO_4_·2H_2_O was used in all experiments to clarify the migration behavior of Ca^2+^ in the Tyr-mediated carbonation process. This choice avoided the complex effects of soluble impurities typically present in industrial waste gypsum, enabling a focused investigation of the underlying mechanism. Analytical-grade KOH, a strong electrolyte, was selected for the alkaline system to ensure efficient dissolution of Tyr and to provide precise stoichiometric control. Tyr and an EDTA standard solution (0.01 mol L^−1^), also of analytical grade, were purchased from Shanghai Macklin Biochemical Co., Ltd., China. All reagents were used as received. Ultrapure water, prepared using a purification system (Merck Chemical Technology Co., Ltd., USA), was employed throughout. CO_2_ gas (purity ≥ 99%) was supplied by Lanzhou Hongli Gas Co., Ltd., China.

### 2.2. Methods

#### 2.2.1. Optimization of Ca^2+^ leaching in the Tyr–KOH–H_2_O system using RSM

The solubility of CaSO_4_·2H_2_O in the Tyr–KOH–H_2_O system was investigated by examining key governing factors: the molar ratio of Tyr to CaSO_4_·2H_2_O (*α*, mol mol^−1^), the molar ratio of KOH to Tyr (*β*, mol mol^−1^), and the liquid-to-solid ratio (*γ*, mL g^−1^). Preliminary ranges for these variables were determined *via* single-factor experiments. A Box–Behnken design (Table S1) was then adopted to optimize the Ca^2+^ leaching yield (*φ*, g L^−1^). The dissolution process was performed in a 250 mL conical flask placed in a thermostatic oscillator at 30 °C and oscillated at 220 rpm for 60 min. After dissolution, the solid and liquid phases were separated by vacuum filtration. The Ca^2+^ concentration in the filtrate was determined by EDTA titration. To explore the interaction between Tyr and Ca^2+^, a portion of the filtrate was freeze-dried and analyzed by Fourier-transform infrared (FTIR) spectroscopy. A blank solution containing Tyr and KOH (without CaSO_4_·2H_2_O) was prepared and treated identically as a reference. FTIR spectra were recorded from 4000 to 400 cm^−1^ at a resolution of 1 cm^−1^ using a Nicolet iS 50 spectrometer (Thermo Scientific, USA) with the KBr pellet method. The undissolved solid residue was dried at 80 °C to constant weight, and its crystalline phase was characterized by X-ray diffraction (XRD, D8 Advance, Bruker, USA) using Cu Kα radiation (*λ* = 1.5406 Å) at 30 kV and 10 mA. XRD data were collected over a 2*θ* range of 10° to 80° with a step size of 0.02° and a scan rate of 1° min^−1^.

#### 2.2.2. Tyr-mediated carbonation reaction

A 150 mL aliquot of the calcium-rich filtrate, obtained under the RSM-optimized leaching conditions, was transferred to a 250 mL three-necked round-bottom flask for carbonation. CO_2_ was continuously bubbled into the solution through a tube connected to an aerator to ensure efficient gas dispersion, with its flow rate controlled by a mass flow controller. The mixture was magnetically stirred at 500 rpm to maintain homogeneity, and the temperature was regulated using a high-precision thermostatic water bath. The specific experimental conditions are listed in Table 1.

**Table 1:** Conditions employed for the carbonation reactions

No.	CO_2_ flow rate (mL min^−1^)	Temperature (°C)	Time (min)
1	100	30	60
2	100	40	60
3	100	50	60
4	100	60	60
5	50	30	60
6	80	30	60
7	100	30	60
8	150	30	60
9	200	30	60
10	150	30	10
11	150	30	20
12	150	30	30
13	150	30	40
14	150	30	50
15	150	30	60

**Fig. 1:**
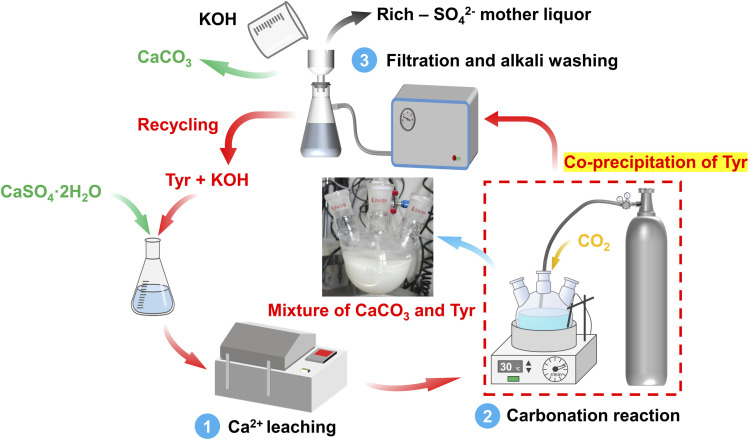
Schematic diagram of the leaching–carbonation process with recyclable Tyr.

The pH was monitored in real-time throughout carbonation using a calibrated pH meter (DZS-708T, INESA Scientific Instrument Co., Ltd., China) to track the reaction progress. Upon completion, the precipitate (primarily CaCO_3_ and co-precipitated Tyr) was separated by vacuum filtration. It was then washed with a KOH solution (prepared at the same concentration as the initial leaching solution) to redissolve Tyr. The washed precipitate was dried at 80 °C to constant weight and characterized by scanning electron microscopy (SEM, Sigma 300, Carl Zeiss AG, Germany), thermogravimetric analysis (TGA, TGA/DSC 3+, Mettler Toledo, Switzerland), particle size distribution analysis (Mastersizer 3000, Malvern Panalytical, UK), and XRD. SEM was conducted at an accelerating voltage of 5 kV. TGA was performed under a nitrogen atmosphere at a heating rate of 10 °C min^−1^ from 30 to 950 °C. The alkaline wash solution containing redissolved Tyr was recycled for five consecutive leaching–carbonation cycles. Before each new cycle, lost Tyr was replenished to restore the pH of the initial alkaline leaching solution. A schematic of the overall experimental procedure is presented in Fig. 1.

Key performance indicators, including the Ca^2+^ leaching yield (*φ*, g L^−1^), carbonation efficiency (*ω*, %), and Tyr recovery efficiency (*η*, %), were calculated using eqn (1)–(3), respectively.

Ca^2+^ leaching yield:1
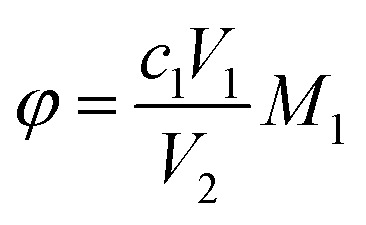


Carbonation efficiency:2
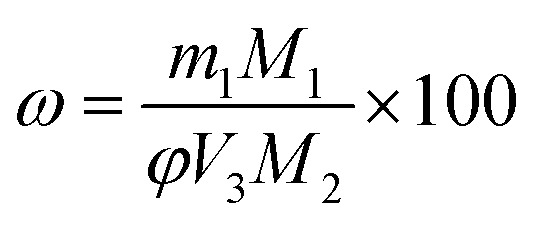


Tyr recovery efficiency:3
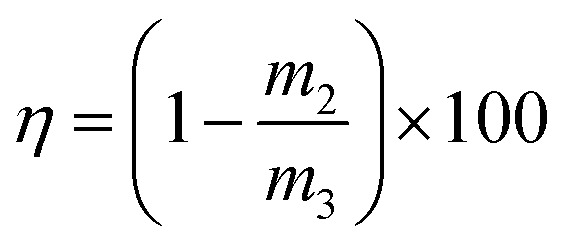
where *c*_1_ (mol L^−1^) and *V*_1_ (mL) are the concentration and volume of the EDTA standard solution, respectively; *V*_2_ (mL) is the volume of filtrate taken for titration (1 mL); *V*_3_ (L) is the total volume of filtrate; *M*_1_ is the molar mass of CaSO_4_·2H_2_O (172.17 g mol^−1^); *M*_2_ is the molar mass of CaCO_3_ (100.09 g mol^−1^); *m*_1_ (g) is the mass of the residue after alkaline washing; *m*_2_ (g) is the mass of Tyr replenished before each cycle; and *m*_3_ (g) is the mass of Tyr added in the first leaching step.

## 3. Results and discussion

### 3.1. Optimization of Ca^2+^ leaching conditions in the Tyr–KOH–H_2_O system

**Fig. 2:**
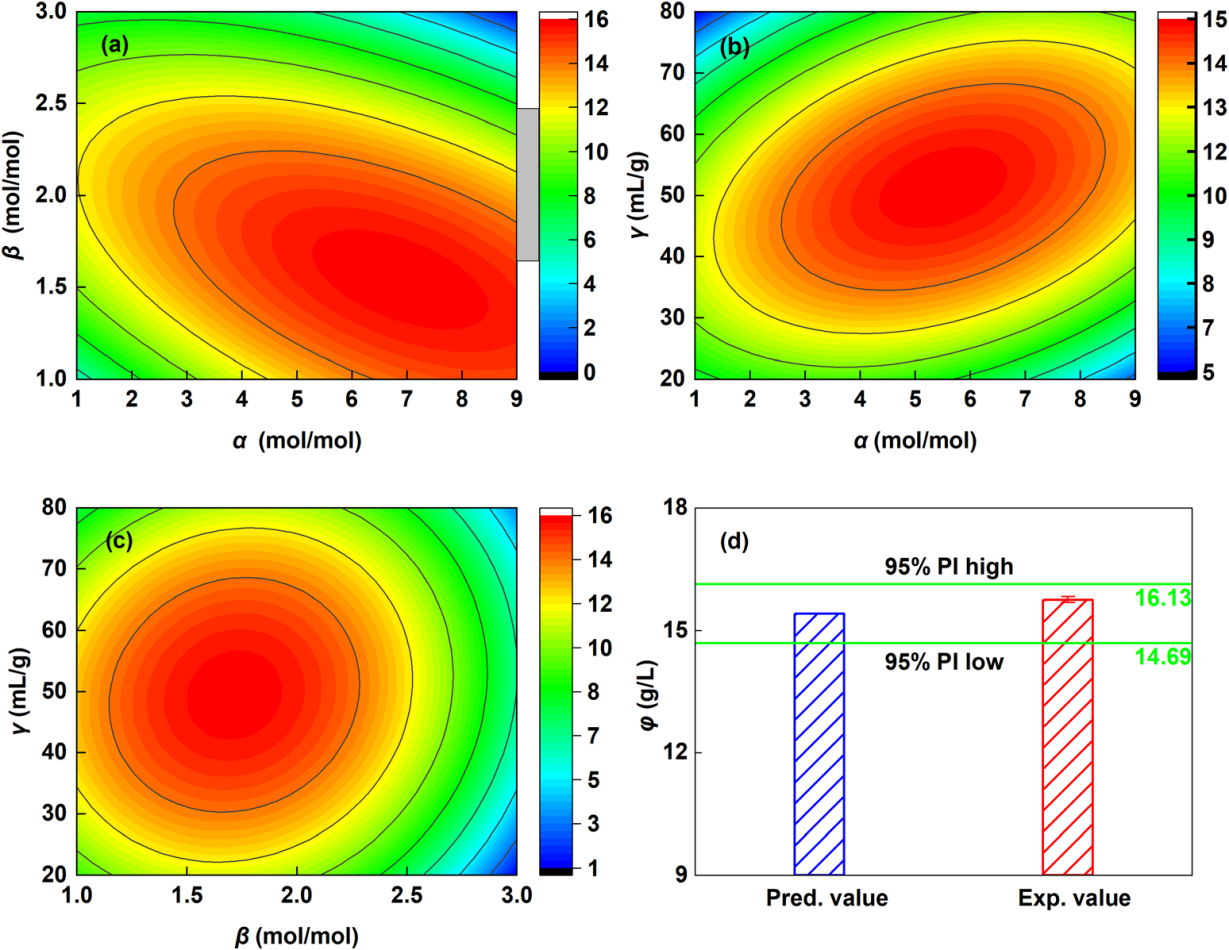
(a–c) Contour plots illustrating the interaction effects among the studied factors; (d) validation experiments performed under the optimized conditions.

The analysis of variance (ANOVA) confirmed the statistical significance of the established model (eqn (S1)) for the Ca^2+^ leaching yield (*φ*). As detailed in Table S2, the model exhibited a high *F*-value of 108.96 and a highly significant *p*-value (<0.0001). The contour plots (Fig. 2a–c) illustrate the interaction effects of the factors on *φ*, revealing distinct peaks within specific operational ranges. As summarized in Table 2, the model predictions agree well with the experimental data across the leaching conditions, with a maximum deviation of 7.91% when data groups with *β* = 3 are excluded. The optimal leaching conditions derived from the model were *α* = 5.51, *β* = 2.00, and *γ* = 51.39 mL g^−1^, corresponding to a predicted maximum *φ* of 15.41 g L^−1^. Validation experiments performed in triplicate (Fig. 2d) showed that all results fell within the 95% prediction interval of the model, further confirming its reliability. In summary, the complexation between Tyr and Ca^2+^ substantially enhanced the solubility of CaSO_4_·2H_2_O, achieving a value over six times greater than in pure water (∼2.6 g L^−1^ at 30 °C).

**Table 2:** Experimental and predicted values of the Ca^2+^ leaching yield (*φ*) under different leaching conditions

No.	*α* (mol mol^−1^)	*β* (mol mol^−1^)	*γ* (mL g^−1^)	*φ* (g L^−1^)		Deviation (%) Abs. value
				Exp. value	Pred. value	
1	9.00	2.00	20.00	6.02	6.25	3.74
2	9.00	1.00	50.00	14.51	14.94	2.98
3	1.00	3.00	50.00	8.03	7.50	6.64
4	9.00	2.00	80.00	11.67	11.17	4.33
5	5.00	3.00	20.00	0.77	0.96	24.03
6	5.00	1.00	80.00	7.80	7.50	3.88
7	1.00	2.00	20.00	9.69	10.02	3.42
8	1.00	1.00	50.00	5.10	5.54	8.69
9	5.00	3.00	80.00	2.26	2.94	29.87
10	5.00	2.00	50.00	15.16	15.32	1.04
11	9.00	3.00	50.00	0.69	0.15	78.99
12	1.00	2.00	80.00	5.62	5.34	4.96
13	5.00	2.00	50.00	15.65	15.32	2.13
14	5.00	1.00	20.00	10.03	9.24	7.91
15	5.00	2.00	50.00	15.94	15.32	3.91
16	5.00	2.00	50.00	15.14	15.32	1.17
17	5.00	2.00	50.00	14.96	15.32	2.39

**Fig. 3:**
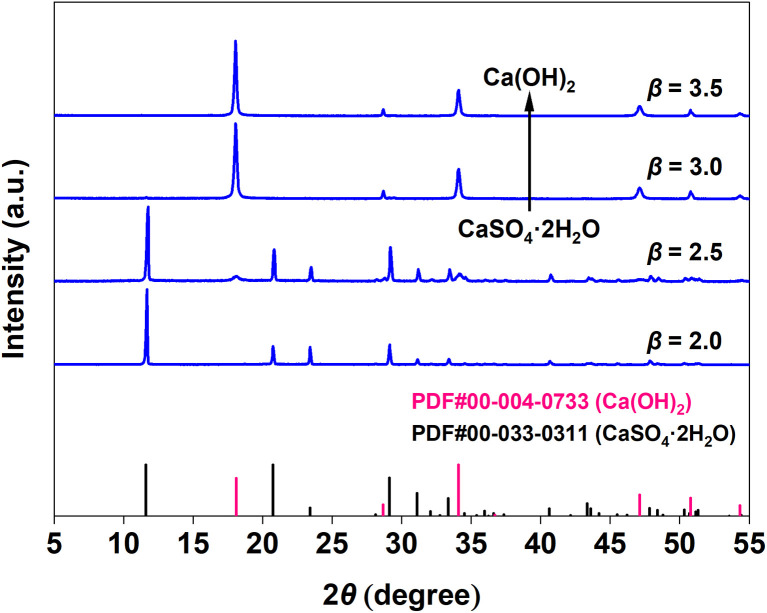
XRD patterns of the residual solid phases obtained after leaching CaSO_4_·2H_2_O at different *β* values.

Response surface analysis indicated that factor *β* (KOH/Tyr molar ratio) exerted the most significant influence on the Ca^2+^ leaching yield. To investigate this effect in detail, a single-factor experiment was conducted. The XRD patterns of the solid residues obtained after leaching CaSO_4_·2H_2_O at different *β* values are presented in Fig. 3. When *β* was below 2.5, the dominant crystalline phase remained CaSO_4_·2H_2_O. With a further increase in *β*, however, the phase converted to Ca(OH)_2_ due to the excess OH^−^. This transformation aligns with the reaction stoichiometry: the molecular structure of Tyr contains one carboxyl and one phenolic hydroxyl group, which react with KOH in a 1 : 2 molar ratio. Once *β* exceeds this stoichiometric point, the surplus OH^−^ reacts with CaSO_4_·2H_2_O to form the less soluble Ca(OH)_2_.^23^ The significant deviation observed at *β* = 3 can therefore be attributed to this phase transformation under highly alkaline conditions.

**Fig. 4:**
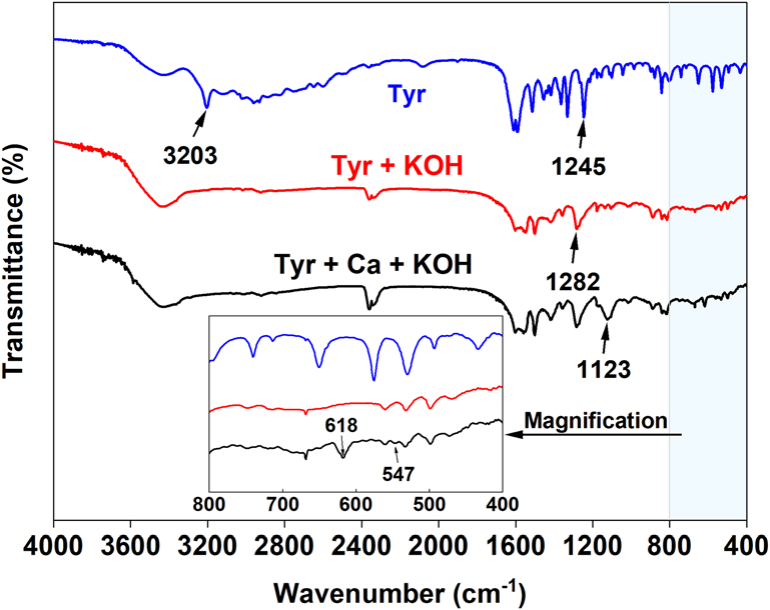
FTIR spectra of the freeze-dried solid from filtrate, blank sample, and analytical-grade Tyr.

The speciation of Tyr is strongly pH-dependent. Based on its dissociation constants (Table S3),^24^ the distribution of its various forms as a function of pH was calculated (Fig. S1). According to acid-base proton theory, in a strongly alkaline KOH solution (pH > 11), the zwitterionic form (Tyr^0^) donates protons from both the α-amino (–NH_3_^+^) and phenolic hydroxyl groups, yielding the anionic species Tyr^−^ and Tyr^2−^. This transformation is corroborated by the FTIR spectra shown in Fig. 4. Compared with analytical-grade Tyr, the blank sample (Tyr + KOH) exhibits the following spectral changes: the characteristic O–H stretching vibration of the phenolic hydroxyl at 3203 cm^−1^ and the N–H stretching vibrations of the –NH_3_^+^ group around 3000 cm^−1^ disappear. Concurrently, the C–O stretching vibration of the phenolic hydroxyl shifts from 1245 cm^−1^ to 1282 cm^−1^. These observations confirm that Tyr exists predominantly as the Tyr^2−^ anion in the leachate when the KOH/Tyr molar ratio (*β*) equals 2. Further comparison between the blank sample and the freeze-dried solid from the filtrate (Tyr + Ca + KOH) reveals an additional peak at 1123 cm^−1^, assigned to the antisymmetric stretching vibration of SO_4_^2−^.^25^ In the magnified low-frequency region (800–400 cm^−1^), two new absorption bands emerge at 547 cm^−1^ and 618 cm^−1^. These bands can be attributed to the stretching vibrations of Ca–O and Ca–N coordination bonds,^26^ indicating the formation of a Ca^2+^–Tyr complex intermediate.

### 3.2. Characterization of the carbonation reaction process involving Tyr

#### 3.2.1. Effect of temperature

**Fig. 5:**
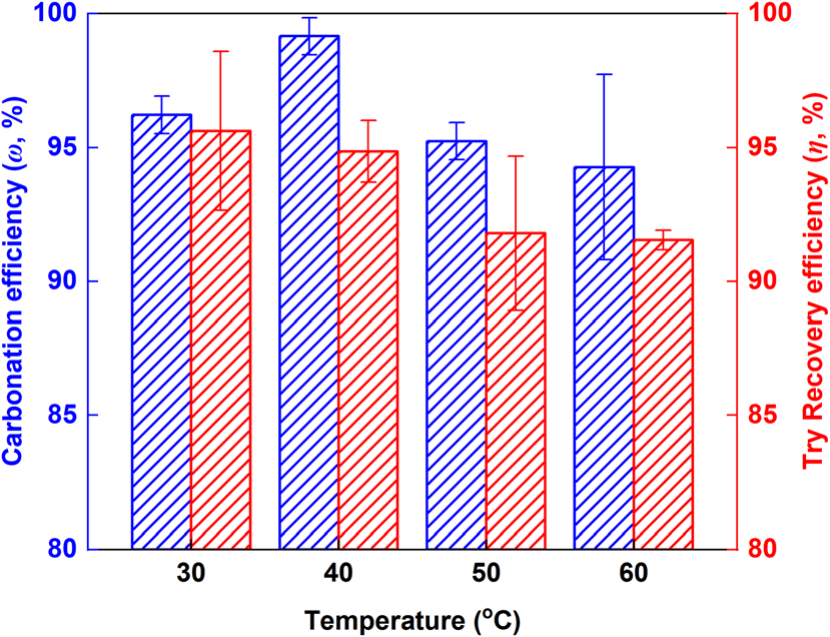
Variation of carbonation efficiency (*ω*) and Tyr recovery efficiency (*η*) with temperature.

Fig. 5 illustrates the influence of temperature on the carbonation efficiency (*ω*) and Tyr recovery efficiency (*η*) under a fixed CO_2_ gas flow rate of 100 mL min^−1^ and a reaction time of 60 min. Over the range of 30 to 60 °C, *ω* varied by less than 5%, indicating a minor dependence on temperature. This behavior is attributed to the high initial pH (∼12), which promotes rapid CO_2_ chemical absorption, resulting in an excess of CO_3_^2−^ over HCO_3_^−^. Although elevated temperatures thermodynamically disfavors the exothermic carbonation and promotes CO_2_ desorption,^27^ the precipitation reaction between Ca^2+^ and CO_3_^2−^ proceeds at a rate significantly higher than that of CO_2_ desorption. Consequently, *ω* did not decrease markedly. In contrast, *η* exhibited a slight decline with increasing temperature, consistent with the higher solubility of Tyr at elevated temperatures.^28^

**Fig. 6:**
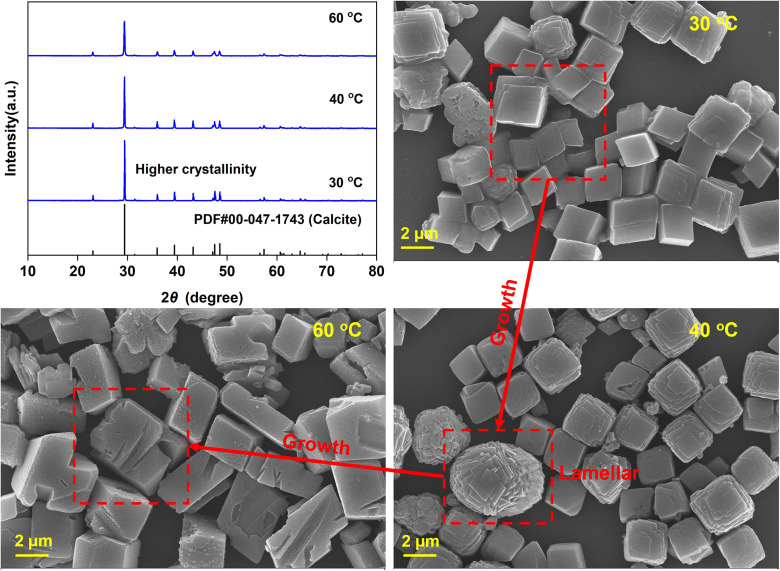
XRD patterns and corresponding SEM images of the CaCO_3_ products formed at different temperatures.

The XRD patterns (Fig. 6) show that a homogeneous calcite product was obtained across the entire temperature range. This contrasts with previous reports using glycine, alanine, or aspartic acid, where vaterite typically persisted as a stable phase.^26,29^ In those systems, deprotonated amino acid anions adsorbed onto initially formed vaterite, inhibiting its transformation to calcite. The predominant formation of calcite in the present system is presumably due to the rapid dissociation of the Ca^2+^–Tyr complex during the initial carbonation stage. Driven by the sharp pH decrease, this dissociation releases free Ca^2+^ ions, which subsequently precipitate directly as calcite—a process with a lower activation energy than that involving chelated calcium.^26^ Furthermore, the crystallinity of CaCO_3_ decreased with increasing temperature. SEM images reveal a slight increase in particle size from 30 to 60 °C, indicating crystal growth. Distinct lamellar steps emerged on crystal surfaces at around 40 °C, which act as active nucleation sites promoting crystal growth *via* a spiral growth mechanism.^30,31^ Therefore, lower temperatures not only improve Tyr recovery efficiency but also favor the formation of well-defined CaCO_3_ crystals.

#### 3.2.2. Effect of CO_2_ flow rate

**Fig. 7:**
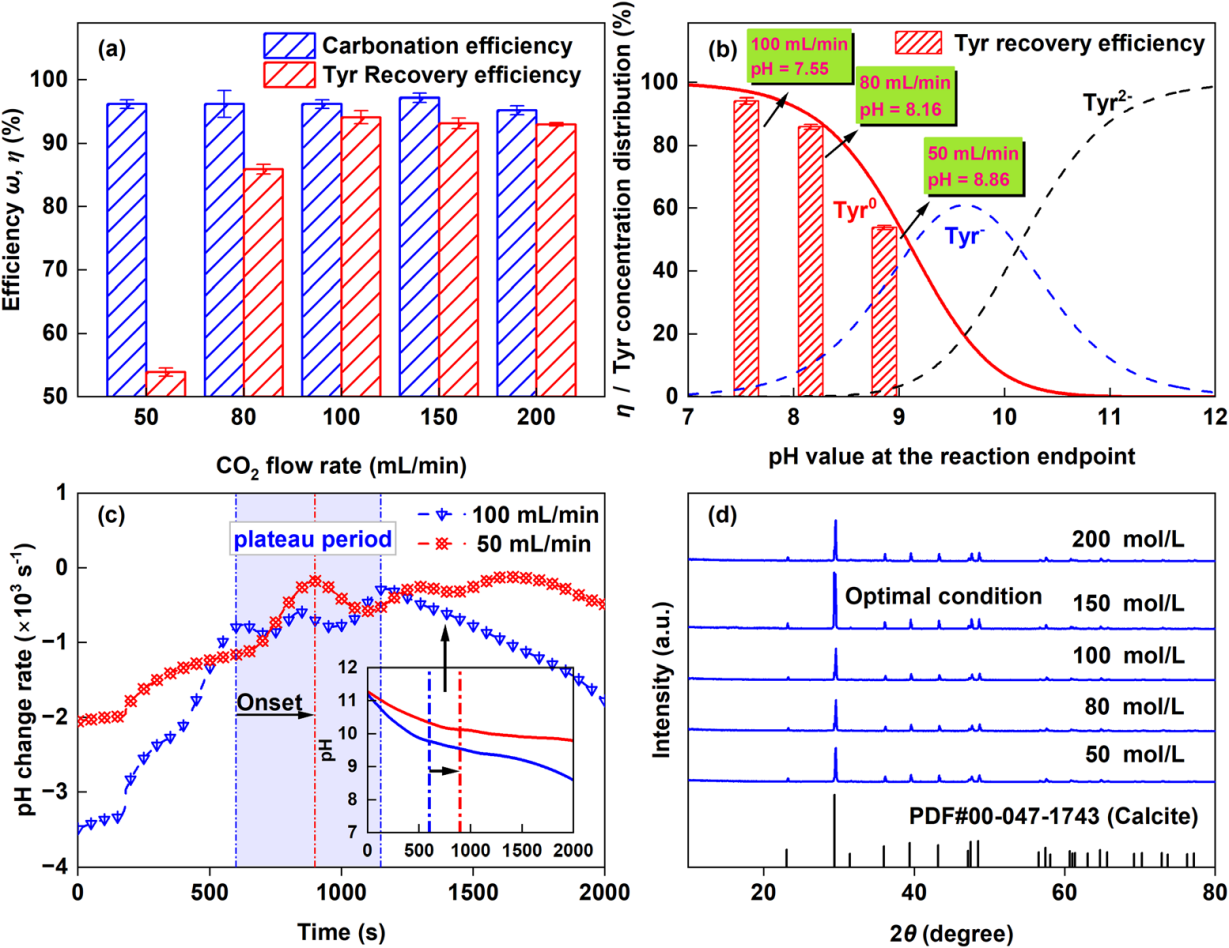
Effects of CO_2_ flow rate on carbonation. (a) Carbonation (*ω*) and Tyr recovery (*η*) efficiencies. (b) Correlation of *η* and Tyr species distribution with pH. (c) Dynamic pH profiles at 50 and 100 mL min^−1^ (d) XRD patterns of the obtained CaCO_3_ products.

Fig. 7a shows the variation of *ω* and *η* with CO_2_ flow rate. The carbonation extent was limited by the dissolved calcium concentration, keeping *ω* relatively stable over the 60 min reaction. *η* increased rapidly as the CO_2_ flow rate rose from 50 to 100 mL min^−1^ and then plateaued at higher flow rates. As shown in Fig. 7b, *η* was strongly correlated with the final solution pH. Increasing the flow rate from 50 to 100 mL min^−1^ lowered the final pH from 8.86 to 7.55. According to the pH-dependent speciation of Tyr (Fig. S1), the proportion of the zwitterionic Tyr^0^ form rises sharply from 66% to 97% over this pH range. Experimentally, *η* followed this trend closely, increasing from 53.89 ± 0.64% to 94.14 ± 1.01%.

Fig. 7c compares the dynamic pH profiles at CO_2_ flow rates of 50 and 100 mL min^−1^. At 100 mL min^−1^, the pH evolution displayed three distinct stages: a rapid but decelerating drop from 11.2 to 9.8 within the first 600 s, a plateau (600–1150 s) with a further slow decline to 9.4, followed by an accelerated decrease. This progression is governed by the kinetics of CO_3_^2−^ formation (Reaction (R1)). In the first stage, rapid dissociation of the Ca^2+^–Tyr complex released abundant Tyr^2−^, whose fraction decreased from >90% to approximately 28% over this pH range (Fig. S1). As a strong proton acceptor, Tyr^2−^ effectively consumed H^+^ generated from Reaction (R1), thereby buffering the initial pH drop and causing the observed deceleration. The plateau corresponds to a period of stable crystal growth, where the rate of CO_3_^2−^ formation was balanced by its consumption through CaCO_3_ crystallization (Reaction (R2)).^32^ In the final stage, the low concentration of free Ca^2+^ led to excess of CO_3_^2−^ formation. Although the dominant Tyr^−^ species still provided buffering, its amino group likely participated in CO_2_ absorption, promoting HCO_3_^−^ formation which exerts a stronger influence on pH and contributes to the accelerated decline.^33^ In contrast, at the lower CO_2_ flow rate of 50 mL min^−1^, the slower rate of CO_3_^2−^ formation delayed the onset of the plateau and extended its duration. XRD patterns (Fig. 7d) show that the diffraction peak intensity of CaCO_3_ initially increased and then decreased with rising CO_2_ flow rate, peaking at 150 mL min^−1^. Consequently, a flow rate of 150 mL min^−1^ was selected to ensure high Tyr recovery and well-crystallized product formation.R1
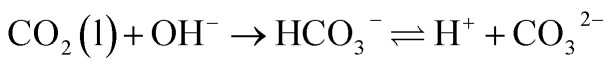

R2 Ca^2+^ + CO_3_^2−^ → CaCO_3_

#### 3.2.3. Effect of reaction time and proposed mechanism

**Fig. 8:**
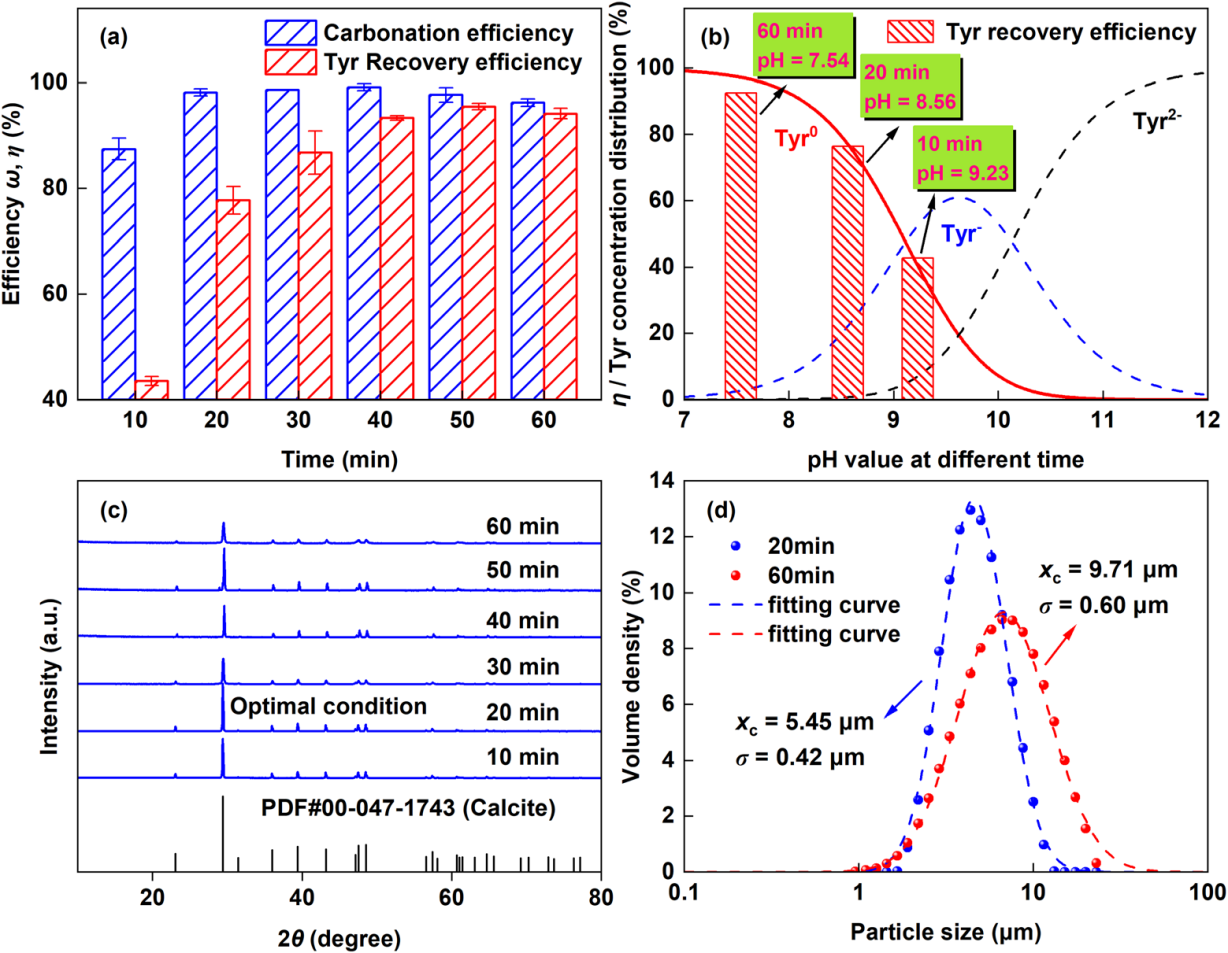
Effects of reaction time on carbonation. (a) Carbonation (*ω*) and Tyr recovery (*η*) efficiencies. (b) Correlation of *η* and Tyr species distribution with pH. (c) XRD patterns of the obtained CaCO_3_ products. (d) Particle size distributions of the CaCO_3_ products obtained at 20 and 60 min.

As shown in Fig. 8a, a marked divergence between *ω* and *η* occurred within the first 10 min: *ω* increased sharply by 87.40%, whereas *η* rose by only 43.49%. *ω* stabilized after 20 min, while *η* required about 40 min to reach a steady state. The rapid CaCO_3_ precipitation observed here contrasts with systems employing Asp, where strong Ca^2+^–Asp complexation prolongs the crystallization induction period and consequently reduces the rate of CaCO_3_ formation.^16,34,35^ The binding affinity between Ca^2+^ and Tyr is weaker than that for Asp.^36^ This weaker interaction promotes rapid Ca^2+^–Tyr complex dissociation as pH drops, releasing free Ca^2+^ for rapid calcite precipitation. Fig. 8b illustrates the rapid increase in the neutral Tyr^0^ species with reaction time, following the pH-dependent distribution. This shift is critical for modulating crystallization and enables efficient Tyr separation. The optimal CaCO_3_ crystallinity was achieved at 20 min (Fig. 8c), coinciding with the maximum *ω*. However, because the precipitation rate exceeded the Tyr recovery rate, a longer reaction time was needed to achieve higher *η*. Particle size distributions (Fig. 8d) were well fitted by a lognormal model (eqn (S2), Table S4). Longer carbonation time increased both the median diameter and distribution width, attributed to particle aggregation and Ostwald ripening.

**Fig. 9:**
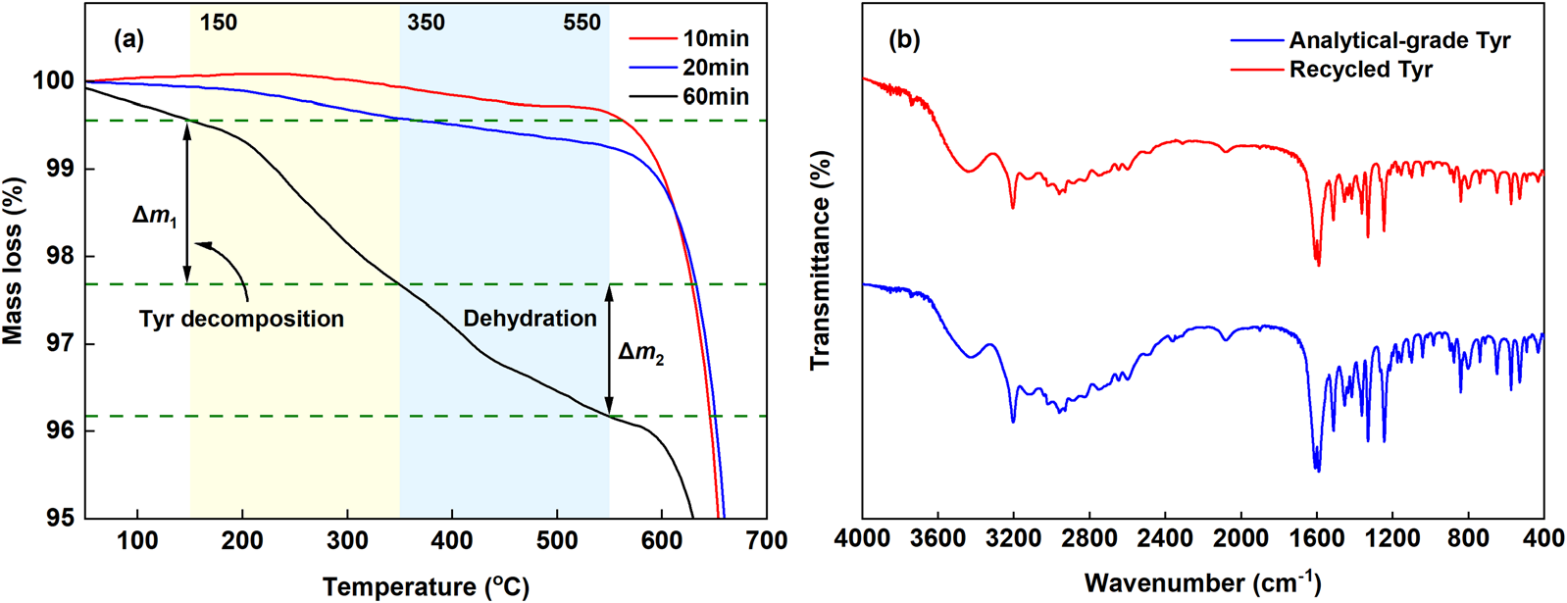
(a) TGA curves of the CaCO_3_ products obtained at different reaction times. (b) FTIR spectra of the recovered Tyr and analytical-grade Tyr.

TGA analysis (Fig. 9a) showed that extending the reaction time from 20 to 60 min significantly increased the total mass loss in the 150–550 °C range from 0.68% to 3.39%. This loss corresponds to the decomposition of Tyr (Δ*m*_1_, 150–350 °C) and the dehydration of crystalline water (Δ*m*_2_, 350–550 °C).^29,37^ During the first 20 min rapid crystallization stage, adsorption of Tyr anions on CaCO_3_ surfaces was insufficient to induce the formation of metastable vaterite. In the later stage, the neutral Tyr^0^ was predominantly incorporated into the crystals, mainly *via* entrapment during dissolution-reprecipitation events associated with Ostwald ripening, identified as the major cause of Tyr loss. The FTIR spectrum of the recovered Tyr (Fig. 9b) closely matches that of analytical-grade Tyr, confirming the high structural integrity of the recycled additive, which was achieved with a maximum recovery efficiency of 95.47% under the optimized reaction conditions.

**Fig. 10:**
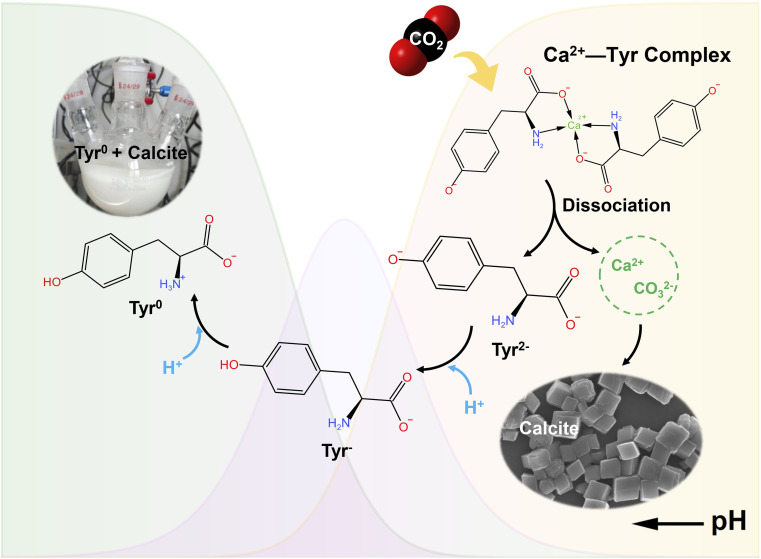
Proposed mechanism for the Tyr-mediated indirect aqueous carbonation of CaSO_4_·2H_2_O.

Based on these findings, a reaction mechanism for the Tyr-mediated indirect aqueous carbonation of CaSO_4_·2H_2_O is summarized (Fig. 10). In the strongly alkaline leaching system, Tyr deprotonates to Tyr^2−^, which complexes with Ca^2+^ to enhance CaSO_4_·2H_2_O dissolution. During subsequent carbonation, the pH drop triggers the rapid complex dissociation, releasing free Ca^2+^ that serves as the calcium source for precipitating calcite-type CaCO_3_. Concurrently, Tyr^2−^ is progressively protonated to Tyr^−^ and finally to the poorly soluble Tyr^0^, which co-precipitates with CaCO_3_. This spontaneous separation, driven by the intrinsic pH swing of the carbonation reaction, eliminates the need for external acid addition or energy-intensive thermal regeneration required in processes using additives such as Asp.

### 3.3. Cycling performance of Tyr over consecutive leaching–carbonation processes

**Fig. 11:**
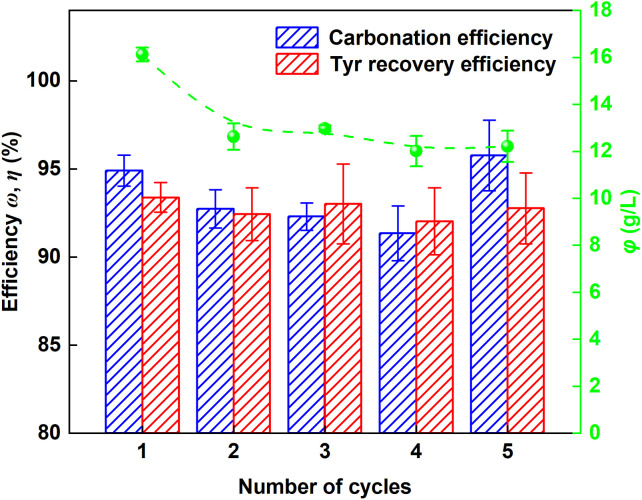
Recycling performance of Tyr over five consecutive leaching–carbonation cycles.

To evaluate the recyclability of Tyr, five consecutive leaching–carbonation cycles were conducted under the optimized condition. As illustrated in Fig. 11, both the carbonation efficiency and Tyr recovery efficiency remained highly stable throughout the cycling tests, averaging 93.41 ± 1.85% and 92.73 ± 0.52%, respectively. In contrast, the Ca^2+^ leaching yield decreased markedly from 16.12 ± 0.30 g L^−1^ in the first cycle to 12.63 ± 0.56 g L^−1^ in the second cycle, representing a reduction of approximately 23%, and then stabilized at an average of 12.46 ± 0.43 g L^−1^ in the following cycles. A similar initial decline in leaching yield has been reported in glycine-mediated indirect carbonation of fly ash.^38^ This reduction may be attributed to the reaction of amino groups with CO_2_, followed by incomplete regeneration of the resulting carbamate intermediates during carbonation. Additionally, SO_4_^2−^ ions adsorb onto the co-precipitates of Tyr and CaCO_3_ during carbonation. These anions are carried into the next leaching cycle with the alkaline wash solution, where the introduced SO_4_^2−^ exerts a common ion effect that suppresses CaSO_4_·2H_2_O dissolution. Importantly, despite this decrease in leaching yield, the Tyr recovery efficiency remained stable throughout the cycles. The effective separation and recovery of Tyr in each cycle is key to preventing the accumulation of SO_4_^2−^ inhibition, which fundamentally distinguishes this process from those that directly recycle the inhibitor-containing mother liquor (*e.g.*, NH_4_Cl or NaCl systems) and enables stable long-term operation.

## 4. Conclusions

This study demonstrates a strategy for the indirect aqueous carbonation of CaSO_4_·2H_2_O using Tyr as an efficient and recyclable leaching additive. The approach is based on utilizing the inherent pH decrease during carbonation to spontaneously precipitate and separate Tyr from the SO_4_^2−^-rich carbonation mother liquor, offering a potential pathway to address the challenge of additive recycling in gypsum carbonation. In a strongly alkaline medium, fully deprotonated Tyr chelates with Ca^2+^, increasing CaSO_4_·2H_2_O solubility by sixfold to 16.09 ± 0.30 g L^−1^ under optimized leaching conditions. During carbonation, the Ca^2+^–Tyr chelates dissociate as the pH drops, releasing free Ca^2+^ for the direct formation of uniform calcite, while neutral Tyr^0^ co-precipitates from the SO_4_^2^-rich carbonation mother liquor. Under the optimized carbonation conditions, the carbonation and Tyr recovery efficiencies exceeded 97% and 95%, respectively. Furthermore, five consecutive leaching–carbonation cycles confirmed the stability of the process.

Future research should focus on the influence of soluble impurities in industrial solid waste gypsum on CaCO_3_ polymorphism and product purity, alongside related process conditions. The underlying cause of the significant leaching yield decline during the initial cycle also requires in-depth clarification. Furthermore, integrating the process with bipolar membrane electrodialysis to recover alkaline media from the spent liquor remains a crucial direction for improving the economic feasibility of the technology.

## Conflicts of interest

The authors declare that they have no known competing financial interests or personal relationships that could have appeared to influence the work reported in this paper.

## Supplementary Material

RA-016-D5RA09358A-s001

## Data Availability

All data generated or analysed during this study are included in this published article. Supplementary information (SI): Tables S1–S4 (Box–Behnken design factors, ANOVA of the calcium leaching model, dissociation constants and isoelectric point of Tyr, and lognormal distribution fit parameters for particle size distribution); Fig. S1 (speciation diagram of Tyr as a function of pH); and eqn (S1)–(S2) (polynomial regression equation and lognormal distribution function). See DOI: https://doi.org/10.1039/d5ra09358a.
