# Supplementary material for: pH-driven spontaneous recovery of tyrosine via co-precipitation in the indirect aqueous carbonation of gypsum
Source: RSC Adv. 2026 Feb 17;16(10):9335–44. doi: 10.1039/d5ra09358a (PMC12910278; doi:10.1039/d5ra09358a)
Supplement: RA-016-D5RA09358A-s001 [file RA-016-D5RA09358A-s001.pdf]

Supplementary Information (SI) for RSC Advances.
This journal is © The Royal Society of Chemistry 2026

pH-Driven Spontaneous Recovery of Tyrosine via Co-precipitation in the Indirect Aqueous Carbonation of Gypsum

Yujie Qu [a], Yuan Gong [a, b, *], Baizhi Wu [a], Chunlei Li [a], Lanying Wang [c], Yi Wang [a, b]

[a] *School of Petrochemical Technology, Lanzhou University of Technology, Lanzhou 730050, PR China*

[b] *Gansu Engineering Research Center for Resource Utilization of New Energy Industry Waste and Waste-Utilized Materials, Lanzhou 730050, PR China*

[c] *Jiuquan Iron and Steel (Group) Co.,Ltd., Jiayuguan 735100, PR China*

*Corresponding author

E-mail address: yuangong@lut.edu.cn (Y. Gong)

Table S1 Factors and levels in the Box-Behnken Design.

level	*α* (mol/mol)	*β* (mol/mol)	*γ* (mL/g)
-1	1.00	1.00	20.00
0	5.00	2.00	50.00
1	9.00	3.00	80.00

Table S2 ANOVA for the calcium leaching model.

Source	Sum of Squares	df	Mean Square	F-value	p-value
Model	457.28	9	50.81	108.96	< 0.0001
α-α	2.48	1	2.48	5.31	0.0547
β-β	82.50	1	82.50	176.91	< 0.0001
γ-γ	0.0882	1	0.0882	0.1891	0.6767
αβ	70.14	1	70.14	150.41	< 0.0001
αγ	23.62	1	23.62	50.65	0.0002
βγ	3.46	1	3.46	7.42	0.0296
α^2	29.04	1	29.04	62.28	< 0.0001
β^2	134.95	1	134.95	289.39	< 0.0001
γ^2	85.03	1	85.03	182.34	< 0.0001
Residual	3.26	7	0.4663		

Lack of Fit	2.60	3	0.8653	5.18	0.0730
Pure Error	0.6684	4	0.1671		
Cor Total	460.55	16			

A polynomial regression equation (Eq. S1) was subsequently developed to correlate φ with the factors α, β, and γ. The goodness-of-fit of the equation is evidenced by the reasonable agreement between the predicted R^2 (0.9075) and the adjusted R^2 (0.9838).

$$\varphi = -0.164\alpha^2 - 5.661\beta^2 - 0.005\gamma^2 - 1.047\alpha\beta + 0.020\alpha\gamma + 0.031\beta\gamma - 20.615 \quad \text{(Eq. S1)}$$

Table S3 Dissociation constants (p*K*) and isoelectric point (p*I*) of Tyr.

	pK_1	pK_2	pK_3	pI
Tyr	2.20	9.11	10.13	5.66

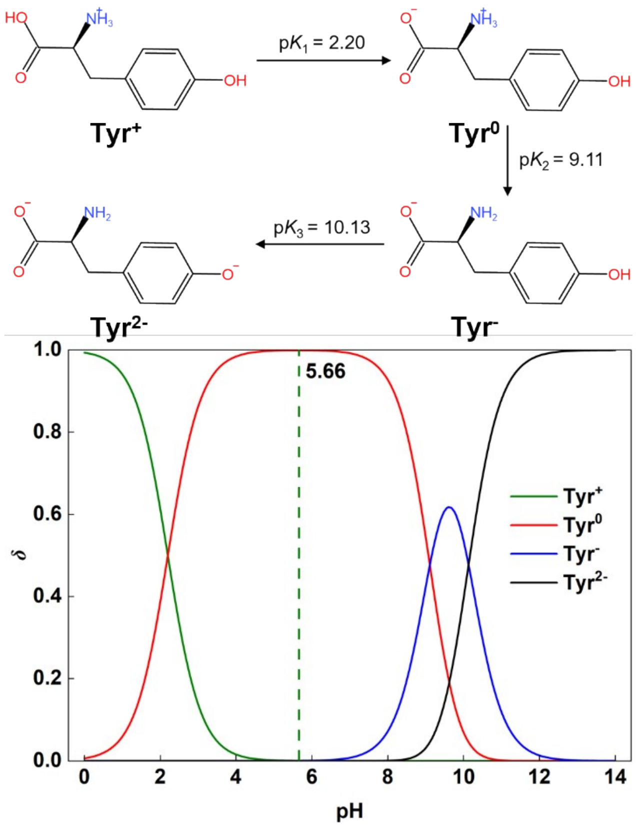

Fig. S1. Speciation diagram of Tyr as a function of pH, illustrating the distribution of its different protonation states.

The PSD of the $CaCO_3$ products obtained at different reaction times was fitted with the lognormal distribution function (Eq. S2). The fitting results are presented in

Table S4.

$$f(x) = \frac{A}{\sqrt{2\pi}\,\sigma x}\exp\left[-\frac{\left(\ln\frac{x}{x_c}\right)^2}{2\sigma^2}\right] \quad \text{(Eq. S2)}$$

In the equation, f(x) is the volume density (%), x is the particle diameter (μm), x_c is the median particle diameter (μm), σ is the standard deviation (dimensionless), and A is the scaling prefactor (dimensionless).

Table S4 Results of the lognormal distribution fit.

Parameters	Reaction time (min)		Unit
	20	60	
A	70.42	113.88	/
x_c	5.45	9.71	μm
σ	0.42	0.60	/
COD (R^2)	0.997	0.996	/
